# Crystal structure of secretory abundant heat soluble protein 4 from one of the toughest “water bears” micro‐animals *Ramazzottius Varieornatus*

**DOI:** 10.1002/pro.3393

**Published:** 2018-04-02

**Authors:** Yohta Fukuda, Tsuyoshi Inoue

**Affiliations:** ^1^ Department of Applied Chemistry, Graduate School of Engineering Osaka University, 2‐1 Yamadaoka Suita Osaka 565‐0871 Japan

**Keywords:** tardigrades, X‐ray crystallography, secretory abundant heat soluble protein, anhydrobiosis

## Abstract

Though anhydrobiotic tardigrades (micro‐animals also known as water bears) possess many genes of secretory abundant heat soluble (SAHS) proteins unique to Tardigrada, their functions are unknown. A previous crystallographic study revealed that a SAHS protein (*Rv*SAHS1) from one of the toughest tardigrades, *Ramazzottius varieornatus*, has a β‐barrel architecture similar to fatty acid binding proteins (FABPs) and two putative ligand binding sites (LBS1 and LBS2) where fatty acids can bind. However, some SAHS proteins such as *Rv*SAHS4 have different sets of amino acid residues at LBS1 and LBS2, implying that they prefer other ligands and have different functions. Here *Rv*SAHS4 was crystallized and analyzed under a condition similar to that for *Rv*SAHS1. There was no electron density corresponding to a fatty acid at LBS1 of *Rv*SAHS4, where a putative fatty acid was observed in *Rv*SAHS1. Instead, LBS2 of *Rv*SAHS4, which was composed of uncharged residues, captured a putative polyethylene glycol molecule. These results suggest that *Rv*SAHS4 mainly uses LBS2 for the binding of uncharged molecules.

## Abstract 2

PDB Code(s): http://firstglance.jmol.org/fg.htm?mol=5z4g

## Introduction

**Figure 1:**
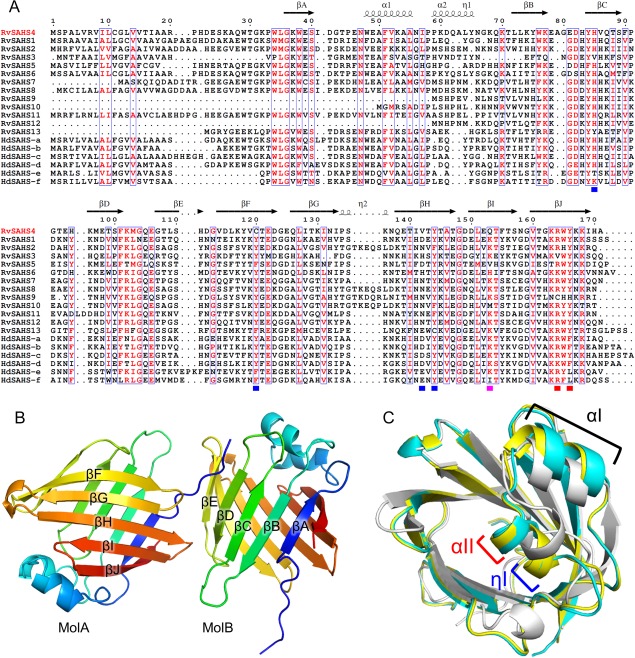
Structure of *Rv*SAHS4. (A) Amino acid sequence alignment. *Rv* and *Hd* means *R*. *varieornatus* and *H*. *dujardini*, respectively. Similar residues are written by red characters and boxed. Red and blue squares under the sequences show residues conserved at LBS1 and LBS2 of SAHS proteins, respectively. A purple square shows the position of K150 in *Rv*SAHS1, which is a part of both LBS1 and LBS2. (B) *Rv*SAHS4 molecules (MolA and MolB) in the asymmetric unit. (C) Comparison of *Rv*SAHS4 with *Rv*SAHS1. MolA and MolB of *Rv*SAHS4 are shown by cyan and yellow cartoons. *Rv*SAHS1 is shown by a white cartoon.

Water is indispensable for all living things. Therefore, severe loss of water results in death for almost all organisms. However, some species of tardigrades, or water bears, can survive extremely desiccated conditions by stopping metabolic processes.^1^, ^2^ This state is called anhydrobiosis. In association with loss of water, anhydrobiotic tardigrades form structures called “tun”, which show a high tolerance to desiccation. They can restart metabolic processes once their tuns are given water.^3^, ^4^ The tun is also tolerant to very high or low temperature,^5^, ^6^ exposure to radiation,^7^, ^8^, ^9^ vacuum,^10^, ^11^ high pressure,^12^, ^13^ and toxic compounds.^14^, ^15^ This resistance of tardigrades is further highlighted by an experiment in which two tardigrades in dehydrated states could survive in the vacuum of outer space for 10 days.^16^ Several research groups have recently launched genomics^17^, ^18^, ^19^, ^20^, ^21^, ^22^ and molecular biological studies^23^, ^24^, ^25^, ^26^ to reveal the molecular basis for anhydrobiosis of tardigrades. Some proteins discovered in these studies are thought to be keys to anhydrobiosis because they have not been found in phyla other than Tardigrada.^23^, ^25^ Secretory abundant heat soluble (SAHS) protein is one of them and is constantly expressed at high levels in *Ramazzottius varieornatus*.^21^ This tardigrade can enter an anhydrobiotic state in a shorter time than other species such as *Hypsibius dujardini*.^24^^,^ 27 Although the expression levels of SAHS proteins are low in active *H*. *dujardani*, they are significantly increased when the tardigrade undergoes anhydrobiosis.^21^ These findings suggest that SAHS proteins play an important role in stress tolerances; however, their functions are unknown. Moreover, it has been recently reported that an anhydrobiotic tardigrade, *Milnesium tardigradum*, does not have SAHS proteins,^22^ making the role of SAHS proteins more enigmatic. One of SAHS proteins from *R. varieornatus* (*Rv*SAHS1) is secreted into the culture medium when it is expressed in human cells; therefore, SAHS proteins are thought to protect extracellular components and/or secretory organelles on anhydrobiosis.^23^ A crystal structure of *Rv*SAHS1 revealed that it has a β‐barrel structure resembling fatty acid binding proteins (FABPs).^28^ Residues found in the β‐barrel of *Rv*SAHS1 were bulky and hydrophilic, while smaller and/or hydrophobic residues are assembled in FABPs. Some FABPs conserve tyrosine and arginine at the binding site for fatty acids (FAs) and *Rv*SAHS1 also has Arg161 and Tyr163, which are superimposed on conserved tyrosine and arginine in FABPs. Moreover, electron density that can be interpreted as an FA molecule is observed near these residues and this site is designated as ligand binding site 1 (LBS1). The analysis of *Rv*SAHS1 also showed electron density that can be interpreted as acetate at the putative ligand binding site 2 (LBS2). The sequence alignment indicates that residues located around LBS1 and LBS2 are conserved among many SAHS proteins [Fig. 1(A)]. However, several SAHS proteins such as *Rv*SAHS4 have different sets of residues around LBS1 and LBS2, which implies differences in functions. Here we analyzed a crystal structure of *Rv*SAHS4 to compare it with *Rv*SAHS1.

## Results and Discussion

The unit cell of the *Rv*SAHS4 crystal contained two *Rv*SAHS4 molecules (MolA and MolB) [Fig. 1(B)]. The *Rv*SAHS4 structure was refined at 1.5 Å resolution. The overall structure of *Rv*SAHS4 shared a typical FABP fold having an antiparallel β‐barrel composed of 10 β‐strands, and a helix‐turn‐helix lid between βA and βB. Because main chain atoms could not form hydrogen bonds between βD and βE, there was a gap as is found in *Rv*SAHS1 and FABPs.^28^ The N‐terminal region of the adjacent molecule was inserted into this gap in the crystal structure [Fig. 1(B)]. The structure of *Rv*SAHS4 was well superimposed on that of *Rv*SAHS1. Helix αII in the *Rv*SAHS4 structure had only a single turn and shorter than those in FABPs as is observed in *Rv*SAHS1 structures. However, MolB in the *Rv*SAHS4 structure had a 3_10_ helix (η1) following αII and MolA also had a helix‐like structure in the same region [Fig. 1(C)]. Consequently, *Rv*SAHS4 had a longer helical region than that in *Rv*SAHS1. The αII‐η1 and following loop region showed higher diversity in amino acid sequences as compared to other regions [Fig. 1(A)], and tended to have proline, glycine, and tyrosine residues, which often inhibit the formation of α helix.^29^ *B*‐factor values showed that the lid region of *Rv*SAHS4 was highly flexible (Fig. S1, Supporting Information), as is observed in *Rv*SAHS1.^28^ The variety of residues and the flexibility in the lid structure may be related to ligand binding.

**Figure 2:**
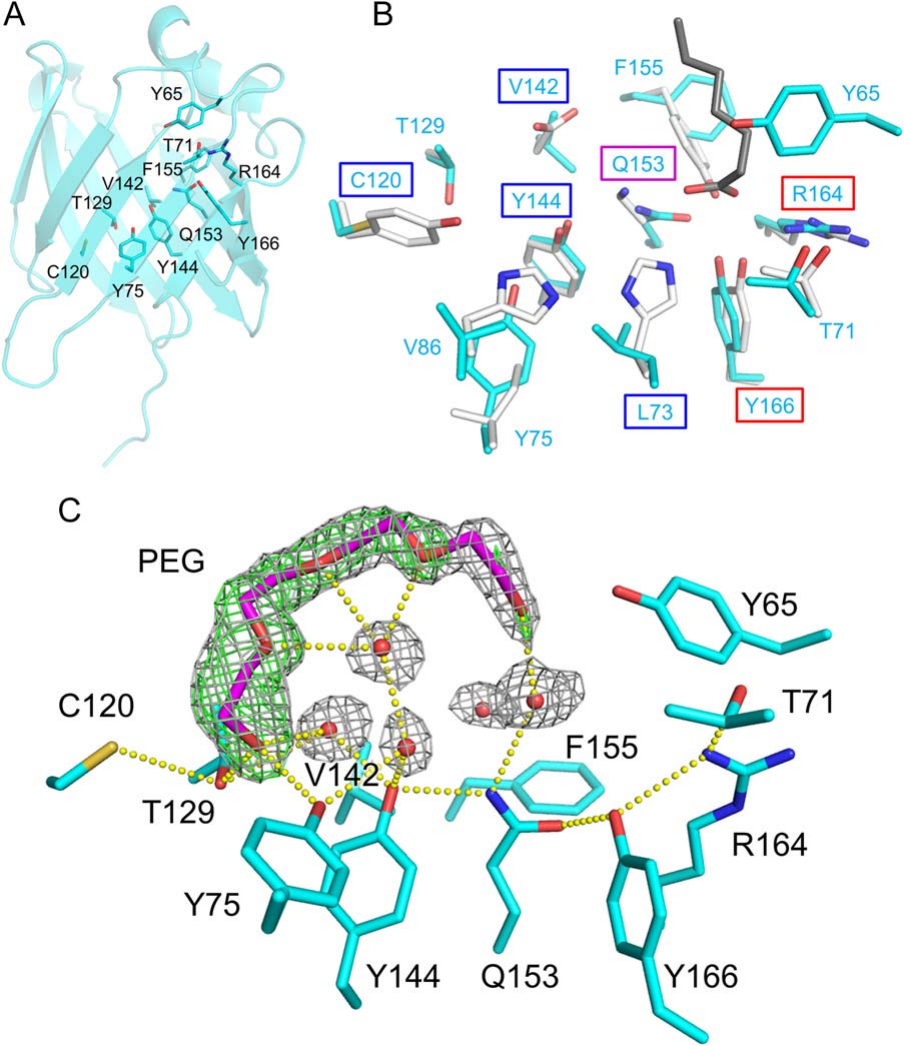
Residues in the β‐barrel of *Rv*SAHS4. (A) LBS1 and LBS2 in *Rv*SAHS4. MolB is shown here. (B) Comparison of LBSs between *Rv*SAHS4 (cyan) and *Rv*SAHS1 (white). Names of residues at LBS1 and LBS2 of *Rv*SAHS4 are boxed by red and blue squares, respectively. A purple square shows the position corresponding to Lys150 in *Rv*SAHS1, which is a part of both LBS1 and LBS2. A putative heptanoic acid molecule found in *Rv*SAHS1 is shown by gray sticks. (C) A tetraethylene glycol molecule in MolB. Water molecules are represented by small red spheres. Carbon atoms of tetraethylene glycol are colored by magenta. 2*mF*_o_‐*DF*_c_ map (1.0σ) and *mF*_o_‐*DF*_c_ omit map (3.0σ) are illustrated by gray and green meshes, respectively. Possible hydrogen bonds are represented by dashed yellow lines.

As was predicted by alignment of amino acid sequences, the interior of *Rv*SAHS4 included residues distinct from those found in *Rv*SAHS1 [Fig. 2(A, B)]. Superimposition of the *Rv*SAHS4 structure on the *Rv*SAHS1 structure revealed that although Arg and Tyr, key residues for the binding of FAs at LBS1 in *Rv*SAHS1, were conserved in *Rv*SAHS4 (Arg164 and Tyr166), Tyr65 positioning on η1 could inhibit the binding of FAs at LBS1 [Fig. 2(B)]. Moreover, residues giving positive charges to LBS1 in *Rv*SAHS1 (His72 and Lys150), which can stabilize negative charges of FA molecules, were replaced by Gln153 and Leu73 in *Rv*SAHS4. While LBS1 in *Rv*SAHS1 possesses an endogenous FA molecule originating from *Escherichia coli* in the crystal structure, no electron density corresponding to an FA molecule was observed around LBS1 in *Rv*SAHS4. Because *Rv*SAHS4 was expressed and purified by the same methods as those for *Rv*SAHS1 and crystallized under a condition (Materials and Methods section) similar to that for *Rv*SAHS1 [100 m*M* HEPES pH 7.8, 150 m*M* MgCl_2_, 1 m*M* ZnSO_4_, 21% v/v polyethylene glycol (PEG) 600, and 5% v/v 1‐butanol or 3% v/v 2‐propanol at 20°C],^28^ the absence of the FA molecule indicates that LBS1 of *Rv*SAHS4 has lower affinity to FAs than that of *Rv*SAHS1. To use LBS1 in *Rv*SAHS4, a dramatic structural change in the helix‐turn‐helix lid may be needed, by which Tyr65 is moved away from LBS1. Residues located at LBS2 in *Rv*SAHS4 were also distinct from those in *Rv*SAHS1. LBS2 in *Rv*SAHS4 was constituted by Cys120, Val142, Tyr144, and Gln153, which respectively correspond to Tyr117, Asp139, Tyr141, and Lys150 in *Rv*SAHS1 [Fig. 2(B)]. Moreover, Tyr75 and Thr129 were located around LBS2 where Leu74 and Ala126 are, respectively, located in *Rv*SAHS1. Cys120 was specific to *Rv*SAHS4 [Fig. 1(A)] and could form a hydrogen bond with Thr129 [Fig. 2(C)]. Slender electron density was observed near Thr129 and Tyr75. Hydrophobic compounds such as FAs, bile acids, and retinoid, which are ligands for FABP family proteins,^30^ could not fit well into the electron density. Therefore, we modeled short PEG molecules contained in the crystallization solution (triethylene glycol in MolA and tetraethylene glycol in MolB) [Fig. 2(C), S2, Supporting Information]. Although PEG molecules seemed to be the best assignment, we do not exclude the possibility that there was another compound such as a disordered FA molecule. The terminal oxygen atom of the PEG molecules could form hydrogen bonds with Thr129, Tyr75, and a nearby water molecule. Other oxygen atoms in the PEG molecules could form hydrogen bonds with water molecules in the β‐barrel. Our observation that LBS1 in *Rv*SAHS4 is blocked by Tyr65 and that PEG molecules were found at LBS2 suggests that *Rv*SAHS4 mainly uses LBS2 to capture ligands. Because neither positively nor negatively charged residues were positioned at LBS2, it may be used to capture uncharged ligands such as alcohols and aldehydes. *Rv*SAHS4 contained several water molecules stabilized by hydrogen bonds around the bottom of the β‐barrel [Fig. 2(C), S2, Supporting Information]. In contrast, there are only one or two water molecules around the β‐barrel bottom of *Rv*SAHS1.^28^ This is because hydrophobic residues in *Rv*SAHS4 (Leu73, Val86, and Phe155) were substituted by residues forming hydrogen bonds with each other in *Rv*SAHS1 (His72, His83, and Tyr152). A hypothesis suggests that *Rv*SAHS1 having the hydrogen bond network being independent of water is stable even under dehydrated conditions.^28^ In line with this thinking, *Rv*SAHS4 containing more water molecules might be more vulnerable to desiccation than *Rv*SAHS1, implying that roles of SAHS proteins may not be limited to responses to dehydration stresses.

## Materials and Methods

### Protein expression and purification

A synthetic gene of *Rv*SAHS4 (*Rv*SAHS4_28–171_) was purchased from GenScript and subcloned into the pET‐28a(+) vector (Novagen). A 6× His tag and a TEV protease site (H_6_‐ENLYFQS) was fused at the N‐terminus of the *Rv*SAHS4 sequence. Therefore, the purified protein contained an extra serine residue at its N‐terminus (The full DNA and amino acid sequences are shown in the text of Supporting Information). The resulting plasmid was used to transform *E*. *coli* strain BL21 Star(DE3). The cells were cultivated in Luria‐Bertani medium supplemented with 100 µg/mL kanamycin at 37°C to an optical density at 600 nm of 0.6. Expression of *Rv*SAHS4 was induced with 0.8 m*M* isopropyl β‐d‐1‐thiogalactopyranoside, and culturing continued for further 19 h at 20°C. Collected cells were sonicated in a lysis buffer (20 m*M* Tris‐HCl pH 8.0, 200 m*M* NaCl, and 5 m*M* imidazole). After the lysate was centrifuged, the supernatant was purified by a HiTrap TALON column (GE Healthcare). Fractions containing *Rv*SAHS4 were collected and dialyzed with TEV protease against a buffer (20 m*M* Tris‐HCl pH 8.0, 200 m*M* NaCl). The dialysate was further purified by a HisTrap Excel column (GE Healthcare) and a HiLoad 16/60 superdex 75 column (GE Healthcare).

### Crystallization

Crystallization was performed by the hanging drop vapor‐diffusion method. *Rv*SAHS4 crystals appeared within 1 week in the presence of 17 mg/mL protein, 50 m*M* HEPES pH 7.8, 1 m*M* ZnSO_4_, and 24% v/v PEG 600 at 20°C. The reservoir solution (400 μL) was poured into 0.5 mL sample cups (Sanplatec), and 1 μL protein solution was mixed with 1 μL reservoir solution on a siliconized cover glass plates. A single plate crystal was peeled off from a petal‐like cluster. Before the crystal was frozen by liquid nitrogen, it was soaked in the crystallization solution supplemented by 20% v/v ethylene glycol.

### Data collection, processing, structure solution, and refinement

X‐ray diffraction experiments were performed on the BL44XU beamline of SPring‐8, Hyogo, Japan, at 100 K using a MX300‐HE detector (Rayonix). The dataset was processed using HKL2000.^31^ The phases were determined by the molecular replacement method with a monomer of *Rv*SAHS1 (PDB code ID: 5XNA) as a search model using Phaser.^32^ Manual model building was performed using Coot.^33^ The program Refmac5^34^ implemented in the CCP4 suite^35^ and phenix.refine^36^ were used for refinement. The final model was checked by MolProbity.^37^ Data collection and processing statistics are summarized in Table 1. The diffraction dataset has been deposited in Integrated Resource for Reproducibility in Macromolecular Crystallography (https://proteindiffraction.org/). The atomic coordinate and structure factor have been deposited in the Protein Data Bank (PDB code ID: 5Z4G).

**Table 1:** Data Collection and Refinement Statistics

	*Rv*SAHS4
Data collection	
Wavelength (Å)	0.90000
Space group	*P*1
*a*, *b*, *c* (Å)	32.8, 41.9, 53.5
α, β, γ (°)	89.97, 80.75, 78.76
Resolution range (Å)	50.0–1.50 (1.53–1.50)^a^
No. of unique reflections	42,396 (2,099)
Completeness (%)	95.9 (95.5)
Redundancy	2.0 (2.0)
*I*/σ(*I*)	23.1 (2.9)
*R*_merge_ (%)	3.6 (26.0)
*R*_r.i.m._ (%)	5.1 (36.8)
CC_1/2_ */*CC* b	(0.818)/(0.949)
Refinement	
Bragg spacings (Å)	50.0–1.50 (1.53–1.50)
Completeness (%)	95.8
No. of reflections	40,319
*R*_work_/*R*_free_ (%)	15.9/20.3
No. of non‐H atoms	
Protein	2543
Water	310
Other	27
R.m.s. deviations	
Bonds (Å)	0.007
Angles (°)	1.224
Average *B* factors (Å^2^)	
Overall	23.8
Protein	23.4
Water	34.7
Other	30.6
Ramachandran plot	
Favored (%)	100
Disallowed (%)	0
PDB code ID	5Z4G

a Data in the parenthesis was calculated based on the highest resolution shell.

b CC_1/2_ and CC* are Pearson correlation coefficient of two half data sets and an estimate of the true CC value, respectively. CC* is calculated from the following equation: CC* = [2CC^1/2^/(1 + CC_1/2_)]^1/2^.

### Sequence alignment

Amino acid sequence alignment was performed by Clustal Omega.^38^ The figure of sequence alignment was generated by ESpript.^39^

## Supporting information

Supporting Information
