# Supplementary material for: Crystal structure of secretory abundant heat soluble protein 4 from one of the toughest “water bears” micro‐animals Ramazzottius Varieornatus
Source: Protein Sci. 2018 Apr 2;27(5):993–9. doi: 10.1002/pro.3393 (PMC5916119; doi:10.1002/pro.3393)
Supplement: Supplementary file 1 — Supporting Information [file PRO-27-993-s001.docx]

**Supporting Information**

**DNA sequence of *Rv*SAHS4_28-171_ in pET28a**

The DNA sequence of *Rv*SAHS4 in pET28a was the following: ATGGGCAGCAGC***CATCATCATCATCATCAC***GAAAACCTGTATTTTCAGTCCCAGTGGACCGGCAAACCGTGGCTGGGCAAGTGGGAAAGCATTGATGGCACCCCGGAAAATTGGGAGGCGTTCGTGAAGGCGGCGAACATCCCGCCGAAAGACCAGGCGCTGTACAACGGCAAGCAAAAAACCCTGCTGAAGTACTGGAAAGAGGCGGGTGAAGATCACTATCACGTTCAGACCAGCTTCCCGGGCACCGAGCACAAGATGGAAACCAGCTTTAAAATGGGTCAAGAGGGCACCCTGAGCCACGACGGTGTGGATCTGAAGTACGTTTGCACCGAGGACGGCGAACAGCTGATCACCAAGATCAACATTCCGAGCAAAAACCAAGAAACCATTGTGACCTATACCGCGACCGGCGACGATCTGGAACAGACCTTCACCAGCAATGGCGTTACCGGCAAGCGTTGGTACAAAAAGATTCATGCGTAA.

The bold italic sequences are the 6×His tag regions. The underline means the TEV protease recognition sequence. The first and last codons, ATG and TAA, are the start and stop codons, respectively.

**Amino acid sequence of purified *Rv*SAHS4_28-171_**

The amino acid sequence of *Rv*SAHS4 purified in this study was the following: MGSSHHHHHHENLYFQ/SQWTGKPWLGKWESIDGTPENWEAFVKAANIPPKDQALYNGKQKTLLKYWKEAGEDHYHVQTSFPGTEHKMETSFKMGQEGTLSHDGVDLKYVCTEDGEQLITKINIPSKNQETIVTYTATGDDLEQTFTSNGVTGKRWYKKIHA.

The underline means the TEV protease recognition sequence. The slash means the TEV protease cleavage site.


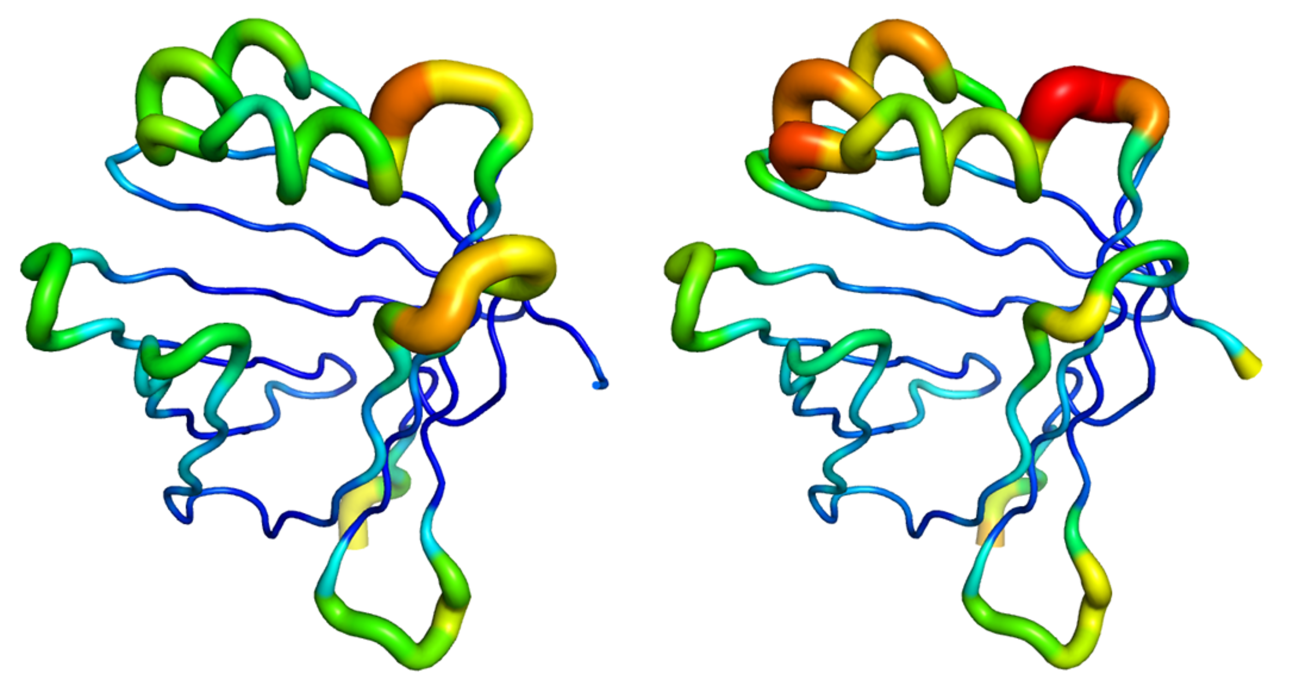


**Figure S1.** Putty representation of *Rv*SAHS4 (left: MolA, right: MolB). Structures are graded in color from lowest (blue) to highest (red) C^α^ *B*-factor values.


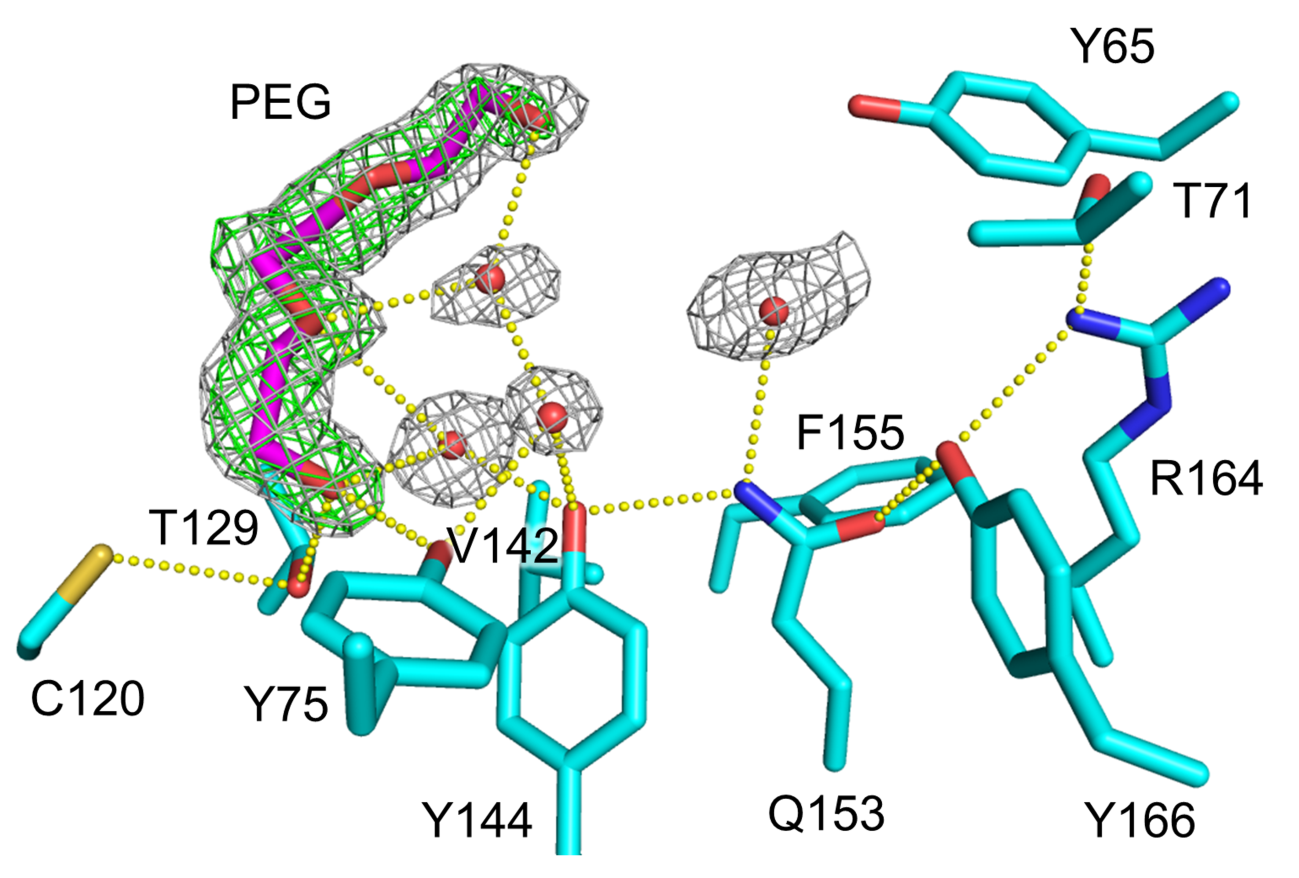


**Figure S2.** A triethylene glycol molecule in MolA. Water molecules are represented by small red spheres. Carbon atoms of triethylene glycol are colored by magenta. 2*mF*_o_-*DF*_c_ map (1.0σ) and *mF*_o_-*DF*_c_ omit map (3.0σ) are illustrated by gray and green meshes, respectively. Possible hydrogen bonds are represented by dashed yellow lines.
